# Recessive *C10orf2* mutations in a family with infantile-onset spinocerebellar ataxia, sensorimotor polyneuropathy, and myopathy

**DOI:** 10.1007/s10048-014-0405-1

**Published:** 2014-05-10

**Authors:** Mi-Hyun Park, Hae-Mi Woo, Young Bin Hong, Ji Hoon Park, Bo Ram Yoon, Jin-Mo Park, Jeong Hyun Yoo, Heasoo Koo, Jong-Hee Chae, Ki Wha Chung, Byung-Ok Choi, Soo Kyung Koo

**Affiliations:** 1 Division of Intractable Diseases, Center for Biomedical Sciences, National Institute of Health, Osong Health Technology Administration Complex 643, Yeonje-ri, Osong-eup, Cheongwon-gun, Chungcheongbuk-do 363-951 South Korea; 2 Department of Neurology, Samsung Medical Center, Sungkyunkwan University School of Medicine, 50 Ilwon-dong Gangnam-Gu, Seoul, 135-710 South Korea; 3 Department of Biological Science, Kongju National University, Gongju, South Korea; 4 Department of Neurology, Ewha Womans University School of Medicine, Seoul, South Korea; 5 Department of Radiology, Ewha Womans University School of Medicine, Seoul, South Korea; 6 Department of Pathology, Ewha Womans University School of Medicine, Seoul, South Korea; 7 Department of Pediatrics, Seoul National University College of Medicine, Seoul, South Korea

**Keywords:** *C10orf2*, Whole exome sequencing (WES), Infantile-onset spinocerebellar ataxia (IOSCA), Neuropathy, Myopathy, Mitochondria

## Abstract

Recessive mutations in *chromosome 10 open reading frame 2* (*C10orf2*) are relevant in infantile-onset spinocerebellar ataxia (IOSCA). In this study, we investigated the causative mutation in a Korean family with combined phenotypes of IOSCA, sensorimotor polyneuropathy, and myopathy. We investigated recessive mutations in a Korean family with two individuals affected by IOSCA. Causative mutations were investigated using whole exome sequencing. Electrophysiological analyses and muscle and nerve biopsies were performed, along with magnetic resonance imaging (MRI) of the brain and lower extremities. Compound heterozygous mutations c.1460C>T and c.1485-1G>A in *C10orf2* were identified as causative of IOSCA. Skeletal muscle showed mitochondrial DNA (mtDNA) deletions. Both patients showed a period of normal development until 12–15 months, followed by ataxia, athetosis, hearing loss, and intellectual disability. Electrophysiological findings indicated motor and sensory polyneuropathies. Muscle biopsy revealed variations in the size and shape of myofibers with scattered, small, and angulated degenerating myofibers containing abnormal mitochondria; these observations are consistent with myopathy and may be the result of mtDNA deletions. Sural nerve biopsy revealed an axonal neuropathy. High-signal-intensity lesions in the middle cerebellar peduncles were correlated with clinical severity, and MRI of the lower legs was compatible with the hypothesis of length-dependent axonal degeneration. We identified novel compound heterozygous mutations of the *C10orf2* gene as the cause of IOSCA with sensorimotor polyneuropathy and myopathy. Signs of motor neuropathy and myopathy were discovered for the first time in IOSCA patients with *C10orf2* mutations. These results suggest that the clinical spectrum of IOSCA caused by *C10orf2* mutations may be more variable than previously reported.

**Electronic supplementary material:**

The online version of this article (doi:10.1007/s10048-014-0405-1) contains supplementary material, which is available to authorized users.

## Introduction

Mutations in nuclear genes involved in mitochondrial DNA (mtDNA) maintenance are increasingly associated with a wide range of clinical phenotypes including encephalopathy, progressive external ophthalmoplegia (PEO), ataxia, myopathy, and Parkinson’s disease [1, 2]. Mutations in DNA polymerase gamma (*POLG*), DNA helicase Twinkle (*C10orf2*), or mitochondrial transcription factor A (*TFAM*) have been linked to the deterioration of mtDNA [3].

*Chromosome 10 open reading frame 2* (*C10orf2*), also known as *Twinkle* or *PEO1*, encodes the mitochondrial helicase Twinkle. Defects in *C10orf2* lead to the accumulation of multiple deletions in the mtDNA of affected tissues and an associated respiratory chain defect [4]. Mutations in the *C10orf2* gene have been shown to play a role in multiple autosomal recessive diseases including infantile-onset spinocerebellar ataxia (IOSCA), hepatocerebral syndrome, and autosomal dominant PEO (adPEO) [5]. However, it remains unclear why some autosomal recessive mutations result in IOSCA while others cause hepatocerebral syndrome.

The clinical symptoms of IOSCA (OMIM# 271245) are characterized by a period of normal development, followed by onset of ataxia, hypotonia, loss of deep tendon reflexes, and athetosis between 9 and 18 months [6]. Later stages of disease are characterized by ophthalmoparesis, sensorineural hearing loss, epilepsy, and primary hypogonadism in female patients. Electromyographic findings are characterized by axonal sensory neuropathy; however, no reports exist describing an IOSCA patient harboring motor neuropathy [5–7]. Although adPEO patients with *C10orf2* mutations usually demonstrate mtDNA deletions in muscle [8, 9], IOSCA patients show no signs of mtDNA deletion. Muscle biopsies of IOSCA patients revealed only nonspecific fiber-type groupings [5–7, 10]. In contrast to the more well-described adPEO, a limited number of reports have been written on IOSCA.

We report the clinical, histopathological, and genetic defects observed with IOSCA, sensorimotor polyneuropathy, and myopathy, which were associated with novel compound heterozygous mutations in *C10orf2*.

## Materials and methods

### Subjects

**Fig. 1:**
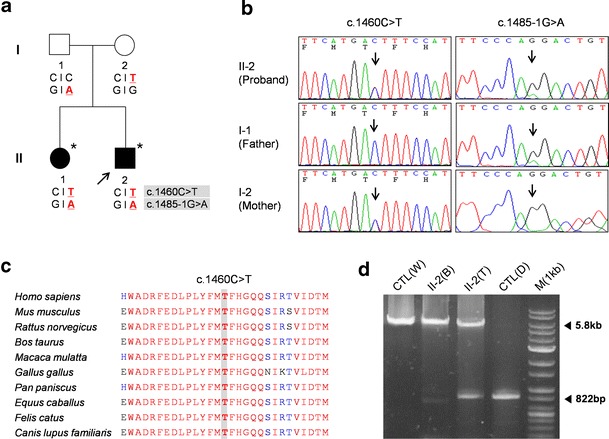
Pedigree, sequencing of *C10orf2* gene mutations, and mtDNA deletions in a Korean family. **a** Pedigree of family FC417. *Open symbols* represent unaffected individuals and *filled symbols* affected individuals. Proband is indicated by an *arrow. Asterisks* indicate individuals whose DNA was used for exome sequencing. *C10orf2* sequences are indicated below each family member. **b** Sequencing chromatograms of *C10orf2* mutations. *Vertical arrows* indicate the mutation site. **c** Conservation of the amino acid sequence in the p.T487I mutation region. The mutation site is highly conserved among vertebrate species: *H. sapiens*, NP_068602.2; *M. musculus*, NP_722491.2; *R. norvegicus*, NP_001101069.1; *B. taurus*, NP_001091933.1; *M. mulatta*, NP_001181370.1; *G. gallus*, NP_001026515.1; *P. paniscus*, XP_003825527.1; *E. caballus*, XP_001499990.1; *F. catus*, XP_003994378.1; and *C. lupus familiaris*, XP_003433659.1. **d** Identification of a 4,977-bp common deletion in mtDNA. The 4,977-bp large deletion (m.8470_13446del4977) was detected using the long template PCR method. *CTL(W)*, wild type; *CTL(D)*, common deletion in adPEO; *II-2(B)*, DNA from the blood of patient II-2; *II-2(T)*, DNA from the vastus lateralis muscle; and *M(1kb)*, 1-kb DNA ladder

This study examined four members of a Korean family (family ID: FC417), two of whom were affected by IOSCA (Fig. 1a). Phenotypic analysis of this family suggested an autosomal recessive mode of inheritance as both children exhibited symptoms of the disease, while both parents were unaffected. No affected individuals were identified among close relatives of the family. Written informed consent was obtained from all participants and from a parent of the participants younger than 18 years of age, according to the protocol approved by the Institutional Review Board of Ewha Womans University, Mokdong Hospital, and the Korea National Institutes of Health (KNIH).

### Clinical and electrophysiological assessments

Two independent neurologists evaluated the patients by recording a detailed history of motor and sensory impairments, deep tendon reflexes, and muscle atrophy. Flexor and extensor muscle strength were assessed manually using the Medical Research Council (MRC) scale. To determine physical disability, we used two scales, a functional disability scale (FDS) [11] and a Charcot–Marie–Tooth neuropathy score (CMTNS) [12]. Disease severity was determined for each patient using a nine-point FDS. Sensory impairments were assessed in terms of the magnitude and severity of pain, temperature, vibration, and position perceptions.

Nerve conduction studies (NCSs) were performed using surface electrodes in the median, ulnar, peroneal, tibial, and sural nerves. Motor nerve conduction velocities (MNCVs) of the median and ulnar nerves were determined by providing stimulation at the elbow and wrist while recording compound muscle action potentials (CMAPs) over the abductor pollicis brevis and adductor digiti quinti, respectively. In the same manner, the MNCVs of the peroneal and tibial nerves were determined by stimulation at the knee and ankle while recording CMAPs over the extensor digitorum brevis and adductor hallucis, respectively. Sensory nerve conduction velocities (SNCVs) were obtained over a finger–wrist segment from the median and ulnar nerves by orthodromic scoring; SNCVs were also recorded for sural nerves. Needle electromyography (EMG) was performed on bilateral proximal and distal limb muscles of the upper and lower extremities. Visual evoked potentials and brainstem auditory evoked potentials in two patients (II-1 and II-2) were done.

### Vastus lateralis muscle biopsy

Histopathological analysis of the skeletal muscle was performed on a 21-year-old patient (patient II-2). The muscle biopsy was taken from the left vastus lateralis muscle under local anesthesia. Frozen sections were stained with hematoxylin and eosin and Gomori’s trichrome stain and for nicotinamide adenine dinucleotide (NADH)-tetrazolium reductase (NADH-TR), succinate dehydrogenase (SDH), and cytochrome oxidase (COX). Adenosine triphosphatase (ATPase) activity was assessed under different pH conditions (ATPase at pH 9.4 followed by preincubation at pH 4.6 and 4.3). An ultrastructural analysis was also performed.

### Distal sural nerve biopsy

Histopathological analysis of the distal sural nerve biopsy was performed at 19 years of age (patient II-2). In addition to light microscopic examination, electron microscopic observations were made using specimens fixed in 2 % glutaraldehyde in 0.025 M cacodylate buffer (pH 7.4) and processed for semi-thin and ultra-thin studies. Semi-thin sections were stained with toluidine blue for evaluation by light microscopy. Ultra-thin sections (60–65 nm) were contrasted with uranyl acetate and lead citrate for ultrastructural studies (H-7650, Hitachi, Japan). The density of myelinated fibers (MFs), axonal diameter, myelin thickness, and the g-ratio of MFs were determined from semi-thin transverse sections using a computer-assisted image analyzer (AnalySIS, Soft Imaging System, Germany).

### Magnetic resonance imaging (MRI) of the brain and lower extremities

The brain, thigh, and lower leg of patients II-1 and II-2 were evaluated by MRI using a 1.5-T system (Siemens Vision, Siemens, Germany). Whole brains were scanned using a slice thickness of 5 mm and a 2-mm interslice gap, to produce 16 axial images. The imaging protocol consisted of T2-weighted spin echo (SE) (repetition time (TR)/echo time (TE) = 4,700/120 ms), T1-weighted SE (TR/TE = 550/12 ms), and fluid-attenuated inversion recovery (FLAIR) (TR/TE = 9,000/119 ms; inversion time = 2,609 ms) images. Thigh and lower leg (calf) muscle imaging was performed in axial [field of view (FOV) = 24–32 cm; slice thickness = 6 mm; slice gap = 0.5–1.0 mm] and coronal planes (FOV = 38–40 cm; slice thickness = 4–5 mm; slice gap = 0.5–1.0 mm). The following protocol was used: T1-weighted SE (TR/TE = 570–650/14–20, 512 matrixes), T2-weighted SE (TR/TE = 2,800–4,000/96–99; 512 matrixes), and fat-suppressed T2-weighted SE (TR/TE = 3,090–4,900/85–99, 512 matrixes).

### Whole exome sequencing

Whole exome sequencing was performed for two individuals (II-1 and II-2). Exome capture/enrichment of 44 Mb was performed using the Human SeqCap EZ Human Exome Library v2.0 (Roche/NimbleGen, Madison, WI). Captured DNA was amplified and sequenced on the Solexa GAIIx (for II-1) and HiSeq2000 (II-2) Genome Analyzers (Illumina, San Diego, CA). Paired-end sequences were mapped to the reference human genome (GRCh37, UCSC hg19) using BWA (http://bio-bwa.sourceforge.net/). The variant calling process was run using SAMtools (http://samtools.sourceforge.net/). For functional annotation and genetic filtering, variants were submitted to ANNOVAR (http://www.openbioinformatics.org/annovar/). Variants were filtered using exome data from 35 Korean patients, including data from 29 CMT patients [13, 14] and 6 deaf patients [15], along with Korean-specific variants deposited in the TIARA database [16]. To confirm candidate variants in additional Korean controls, we used exome data from an additional 648 patient samples.

Capillary sequencing was performed to confirm candidate variants. PCR products were sequenced using an ABI 3730 automatic genetic analyzer (Applied Biosystems, Foster City, CA). Mutations were described according to the Human Genome Variant Society (HGVS) nomenclature (http://www.hgvs.org/mutnomen) with nucleotide numbering based on the mRNA sequence (NM_021830.4) of *C10orf2* (http://www.ncbi.nlm.nih.gov/nuccore/NM_021830). Segregation analysis of mutations with disease phenotypes was performed for all family members.

### Mitochondrial DNA analysis

A deletion of 4,977 bp (m.8470_13446del4977) in mtDNA, frequently referred to as the “common deletion,” was tested in DNA extracted from whole blood and the vastus lateralis muscle of patient II-2, using an Expand Long Template PCR System (Roche, Germany). PCR primers covered nucleotides 8,225–8,247 (forward) and 13,707–13,729 (reverse) of the revised Cambridge reference sequence (NC_012920.1).

## Results

### Identification of the novel mutation in the *C10orf2* gene

We performed whole exome sequencing on an autosomal recessive IOSCA family with sensorimotor polyneuropathy and myopathy to identify mutations causally associated with disease onset (Fig. 1a). Exome sequencing results of two individuals are shown in Table 1. A total of 58,262 and 61,340 variants were identified in each of the two individuals, respectively. Of these, a total of 10,714 and 9,543 variants were identified as functionally significant, resulting in missense, nonsense, splice, or indel mutations. After filtering out all dbSNP132 variants, we were able to reduce the numbers of functional variants to 1,231 and 824, respectively, with 365 variants shared between the two individuals. Additional filtering was performed by excluding all variants identified in a cohort of 35 Korean control exomes.

**Table 1:** Exome sequencing results for two affected individuals of family FC417

Patients	II-1	II-2
Mappable yield (bp)	5,206,019,945	5,937,912,039
On-target yield (bp)	3,248,319,559	3,393,707,908
% Coverage of targeted region (44 Mb) >1×	97.9 %	97.0 %
% Coverage of targeted region (44 Mb) >10×	94.0 %	90.9 %
Mean read depth of targeted region	73.8×	77.1×
Mean read depth of called variant ≥5×	58.9×	55.4×
Number of SNPs	50,733	53,119
Number of indels	7,529	8,221
Total variants	58,262	61,340
Total coding, splicing variants	21,084	19,516
Missense, nonsense, splice, indel variants	10,714	9,543
After dbSNP132 filtering	1,231	824
Shared variants between the two individuals	365	
After filtering with Korean control exomes^a^	146	
Genes with compound heterozygous variants	2 (C10orf2 and LILRB1 genes)	

^a^Exome data of 35 Koreans from other reports and the Korean genomes database, TIARA

Based upon these analyses, we were able to identify compound heterozygous variants in two genes, *C10orf2* and *leukocyte immunoglobulin-like receptor, superfamily B* (*LILRB1*) (Table 1). We compared the four variants in *C10orf2* (Chr10:102749617 and Chr10:102750192) and *LILRB1* (Chr19:55143632 and Chr19:55145125) with those identified in the 248 exome dataset. Only two variants in gene *C10orf2* were absent from the 248 exome dataset (Supplementary Table 1); these variants were also absent in a larger collection of in-house exome data (*n* = 400). We confirmed that both *C10orf2* heterozygous mutations c.1460C>T (p.T487I) and c.1485-1G>A showed complete segregation with the disease phenotype within the family (Fig. 1a, b). The patients’ father carried a c.1485-1G>A splicing-site mutation, while the mother carried the c.1460C>T missense mutation. The p.T487I mutation was located at a highly conserved location (Fig. 1c) and was predicted to be damaging, according to in silico analyses using SIFT, PolyPhen-2, and MUpro (Table 2). As several mutations in *C10orf2* have been implicated in recessive IOSCA [6–8], we therefore concluded that the two novel compound heterozygous mutations in *C10orf2* described here were the underlying cause of IOSCA in this family.

**Table 2:** *C10orf2* heterozygous mutations associated with IOSCA with sensorimotor polyneuropathy and myopathy

Gene	Mutation		In controls	In silico analysis		
	Nucleotide	Amino acid		SIFT	PolyPhen-2	MUpro
*C10orf2*	c.1460C>T	p.T487I	0/648	0.02^a^	1.00^a^	−0.566^a^
	c.1485-1G>A	Splicing acceptor	0/648	–	–	–

^a^Values indicate prediction of significant effect on protein structure or function

### mtDNA deletion in muscle

As mutations in *C10orf2* are associated with deletions in mtDNA, we examined the mtDNA of patient 1 (II-2); this revealed a large heteroplasmic common deletion of 4,977 bp (m.8470_13446del4977) in skeletal muscle, whereas few or no deletions were identified in blood mtDNA (Fig. 1d).

### Clinical manifestations

**Fig. 2:**
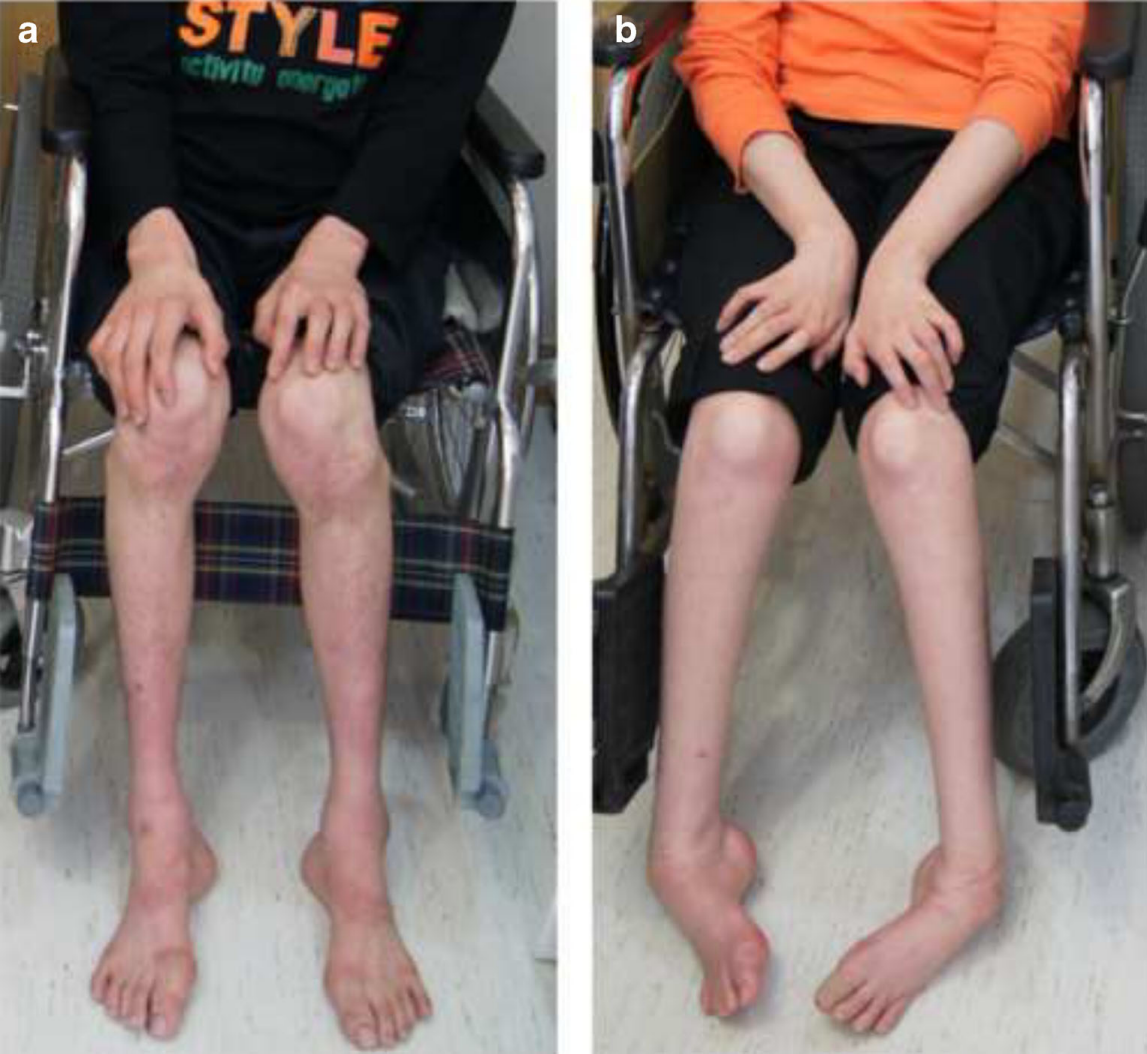
Images of patients II-2 (**a** 21 years of age) and II-1 (**b** 23 years of age). **a** Patient II-2 was unable to walk and has been wheelchair-bound since 5 years of age. Moderate-to-severe atrophies of the lower limb muscles and high-arched feet were evident. **b** Patient II-1 was wheelchair-bound by 7 years of age. Atrophies of the lower leg and hand muscles were prominent. Substantial ankle joint contractures and foot deformities were also evident

The clinical features of patients with *C10orf2* mutations are described in Table 3. Patient II-2 (Fig. 2a, proband) was the second child of healthy, non-consanguineous Korean parents. He was born at full term, and the prenatal and neonatal history was unremarkable. Early motor milestones were not significantly delayed until 1 year of age, when his parents noticed that he had become clumsy and spoke less often than before. He was in a wheelchair by the age of 14 years. At age 17, we performed MRI of the brain as the result of an ataxia. By 20 years of age, ataxia had become more severe, and a follow-up MRI was performed. Neurological examination at 21 years of age revealed severe distal muscle atrophy, pes cavus, and scoliosis. Symptoms of ophthalmoplegia were not observed. An ophthalmologist examined the patient, including dilated fundus examination, and he confirmed that no optic atrophy was present. Vibration and position senses were more severely disturbed than pain and temperature senses. Deep tendon reflexes were absent in all extremities. FDS and CMTNS scores were 7 and 26, respectively, with both scores classifying this patient as severely disabled. He also exhibited substantial intellectual disability, with an intelligence quotient score of 54. A positron emission tomography (PET) scan of patient II-2 showed decreased metabolic changes throughout the brain. An echocardiogram was normal, and there was no evidence of migraine, seizure, or psychiatric symptoms. An electroencephalogram revealed a normal sleep record without epileptiform discharges. Secondary sex characteristics were well developed, with no signs of hypogonadism. His blood lactate level was 17.0 μmol/L (reference interval 4.5–14.4 μmol/L), pyruvate level was 0.7 μmol/L (reference interval 0.3–0.9 μmol/L), and creatine kinase level was 155 μmol/L (reference value <185 μmol/L), all well within normal ranges. Liver transaminase levels were normal: the AST level was 22 μ/L (reference value <40 μ/L), and the alanine transaminase (ALT) was 27 μ/L (reference value <40 μ/L).

**Table 3:** Comparison of the clinical phenotypes of IOSCA patients with mutations in the *C10orf2* gene

Patients (ethnicity)	Korean	English	Finnish	Turkish
Mutations	[T487I]+[1485-1G>A]	[P83S]+[R463W]	[Y508C]+[Y508C], [Y508C]+[A318T]	[L456V]+[L456V]
Number of patients	2	1	23	2
Onset age (years)	>1	>1	>1	>1
Normal early milestones	Yes	Yes	Yes	Yes
Athetosis	Yes	Yes	Yes	Yes
Areflexia	Yes	Yes	Yes	Yes
Pes cavus	Yes	NA	Yes	Yes
Scoliosis	Yes	NA	Yes	NA
Hearing loss	Yes	NA	Yes	Yes
Psychomotor retardation	Yes	NA	Yes	Yes
Ophthalmoplegia	No	Yes	Yes	Yes
Liver involvement	No	No	Yes	No
Kidney involvement	No	No	No	No
Nerve conduction study	Sensorimotor polyneuropathy	Sensory neuropathy	Sensory neuropathy	Sensory neuropathy
Electromyography	Abnormal	Abnormal	Abnormal	Abnormal
Electroencephalography	Normal	Abnormal	Abnormal^a^	Normal
mtDNA depletion	NA	NA	Brain, liver	NA
mtDNA deletion	Yes	NA	No	NA
Liver enzyme	Normal	Abnormal^b^	Abnormal^c^	Normal
Creatine kinase	Normal	NA	Normal	NA
Lactate	Elevated	Elevated^d^	Normal	Normal
Pyruvate	Normal	NA	Normal	NA
Lactate/pyruvate ratio	45.6, 24.3	NA	NA	NA
Respiratory chain	Normal	Normal	Normal	NA
Brain MRI	Abnormal	Abnormal	Abnormal	Abnormal
Lower extremity MRI	Abnormal	NA	NA	NA
Muscle biopsy	Myopathy, type grouping	Type grouping	Type grouping	Type grouping
Nerve biopsy	Axonal loss	Axonal loss	Axonal loss	NA
References	This study	[7]	[5, 10, 22, 23]	[6]

*NA* not available

^a^Abnormal after epileptic encephalopathy

^b^When her symptoms worsen, liver enzymes were elevated

^c^Abnormal in patients with compound heterozygote mutation

^d^Inconsistently elevated in capillary lactate, while normal in CSF lactate

Patient II-1 (Fig. 2) was the elder sister of patient II-2 and was born at full term after an uneventful pregnancy. Early motor milestones were not significantly delayed until 15 months, when her parents noticed that she had become clumsy. She was first examined at 3 years of age due to ataxia and athetoid movements; however, symptoms of ophthalmoplegia were not observed. An ophthalmologist examined the patient, and he confirmed that no optic atrophy was present. A hearing deficit was found, and by school age, she was completely deaf and began communicating with sign language. She became wheelchair bound by the age of 14 years. When we examined her at 23 years of age, she displayed muscle weakness and atrophy of the bilateral distal muscles of the upper and lower limbs. Loss of sensory and tendon reflexes was similar to those of her younger brother. FDS and CMTNS scores were 7 and 28, respectively, both in the severe disability category. Pes cavus and scoliosis were observed. Like her brother, she also exhibited significant intellectual disability. An echocardiogram was normal, and there was no evidence of migraine, seizure, or psychiatric symptoms. An electroencephalogram revealed a normal sleep record without epileptiform discharges. Secondary sex characteristics were poorly developed, and she never menstruated. Her lactate level was 41.0 μmol/L, pyruvate level was 0.9 μmol/L, and creatine kinase level was 71 μmol/L, all within normal ranges. Liver transaminase levels were normal: the AST level was 23 μ/L, and the ALT level was 38 μ/L. The father (I-1, 50 years of age) and mother (I-2, 47 years of age) were found to be normal by careful clinical and electrophysiological examinations.

### Electrophysiological findings

The electrophysiological features of patients II-1 and II-2 are described in Table 4. NCSs were performed three times, at 19, 20, and 21 years of age for patient II-2, and at 22 years of age for patent II-1. MNCVs of the median, ulnar, peroneal, and tibial nerves were all decreased. In patient II-2, peroneal CMAPs were not elicited, and tibial CMAPs were reduced. Sensory nerve action potentials (SNAPs) of the median, ulnar, and sural nerves were absent in both patients. The interval changes over 3 years in patient II-2 revealed slow disease progression. Needle EMG showed fibrillation and positive sharp waves in the proximal and distal muscles. Delayed visual evoked potentials and brainstem auditory evoked potentials were decreased in both patients.

**Table 4:** Electrophysiological features of IOSCA patients with compound heterozygous *C10orf2* mutations

	Patient II-1		Patient II-2						Normal value
Age at exam (years)	22		19		20		21		
Side	Rt	Lt	Rt	Lt	Rt	Lt	Rt	Lt	
Median nerve									
TL (ms)	3.5	3.3	**4.3**	**4.2**	**4.6**	**4.2**	**4.1**	**4.3**	<3.9
CMAP (mV)	**5.9**	8.8	9.7	7.2	9.6	6.1	11.3	6.2	>6.0
MNCV (m/s)	**48.9**	**48.9**	**48.1**	**47.9**	**47.3**	**42.9**	**47.2**	**43.6**	>50.5
F-wave (ms)	**29.6**	**29.0**	**34.0**	**32.8**	**34.6**	**35.2**	**36.6**	**33.6**	<28.0
Ulnar nerve									
TL (ms)	2.7	2.6	2.9	**3.4**	**3.3**	**3.5**	2.6	**3.1**	<3.0
CMAP (mV)	11.1	8.6	10.1	9.0	10.8	**7.7**	9.9	**7.3**	>8.0
MNCV (m/s)	**48.9**	**47.8**	**49.0**	**47.2**	**45.5**	**43.1**	**45.5**	**45.5**	>51.1
F-wave (ms)	**30.0**	**29.2**	**32.8**	**33.0**	**34.6**	**35.0**	**37.4**	**34.2**	<29.0
Peroneal nerve									
TL (ms)	**5.4**	**5.5**	**A**	**A**	**A**	**A**	**A**	**A**	<5.3
CMAP (mV)	**1.5**	**0.8**	**A**	**A**	**A**	**A**	**A**	**A**	>1.6
MNCV (m/s)	**37.4**	**36.6**	**A**	**A**	**A**	**A**	**A**	**A**	>41.2
F-wave (ms)	**52.2**	**73.8**	**A**	**A**	**A**	**A**	**A**	**A**	<49.0
Tibial nerve									
TL (ms)	2.8	3.3	**7.2**	5.0	4.2	4.4	4.2	4.5	<5.4
CMAP (mV)	**2.7**	**3.9**	**1.5**	**1.1**	**1.3**	**1.0**	**0.8**	**1.0**	>6.0
MNCV (m/s)	**36.6**	**37.9**	**24.2**	**27.3**	**28.7**	**29.4**	**28.1**	**32.0**	>41.1
F-wave (ms)	**56.6**	**49.0**	**75.8**	**A**	**75.6**	**70.4**	**72.2**	**71.0**	<52.1
Median sensory nerve									
SNAP (μV)	**A**	**A**	**A**	**A**	**A**	**A**	**A**	**A**	>8.8
SNCV (m/s)	**A**	**A**	**A**	**A**	**A**	**A**	**A**	**A**	>39.3
Ulnar sensory nerve									
SNAP (μV)	**A**	**A**	**A**	**A**	**A**	**A**	**A**	**A**	>7.9
SNCV (m/s)	**A**	**A**	**A**	**A**	**A**	**A**	**A**	**A**	>37.5
Sural nerve									
SNAP (μV)	**A**	**A**	**A**	**A**	**A**	**A**	**A**	**A**	>6.0
SNCV (m/s)	**A**	**A**	**A**	**A**	**A**	**A**	**A**	**A**	>32.1
H-reflex (ms)	**A**	**A**	**A**	**A**	**A**	**A**	**A**	**A**	<30.2

Bold characters indicate abnormal values. Normal conduction velocities: median motor nerve ≥50.5 m/s; ulnar nerve ≥51.1 m/s; and sural nerve ≥32.1 m/s. Normal amplitudes: median motor nerve ≥6.0 mV; ulnar nerve ≥8.0 mV; and sural nerve ≥6.0 μV

*A* absent potentials, *TL* terminal latency, *CMAP* compound muscle action potential, *MNCV* motor nerve conduction velocity, *SNAP* sensory nerve action potential, *SNCV* sensory nerve conduction velocity, *NP* no potential

### Muscle biopsy findings

**Fig. 3:**
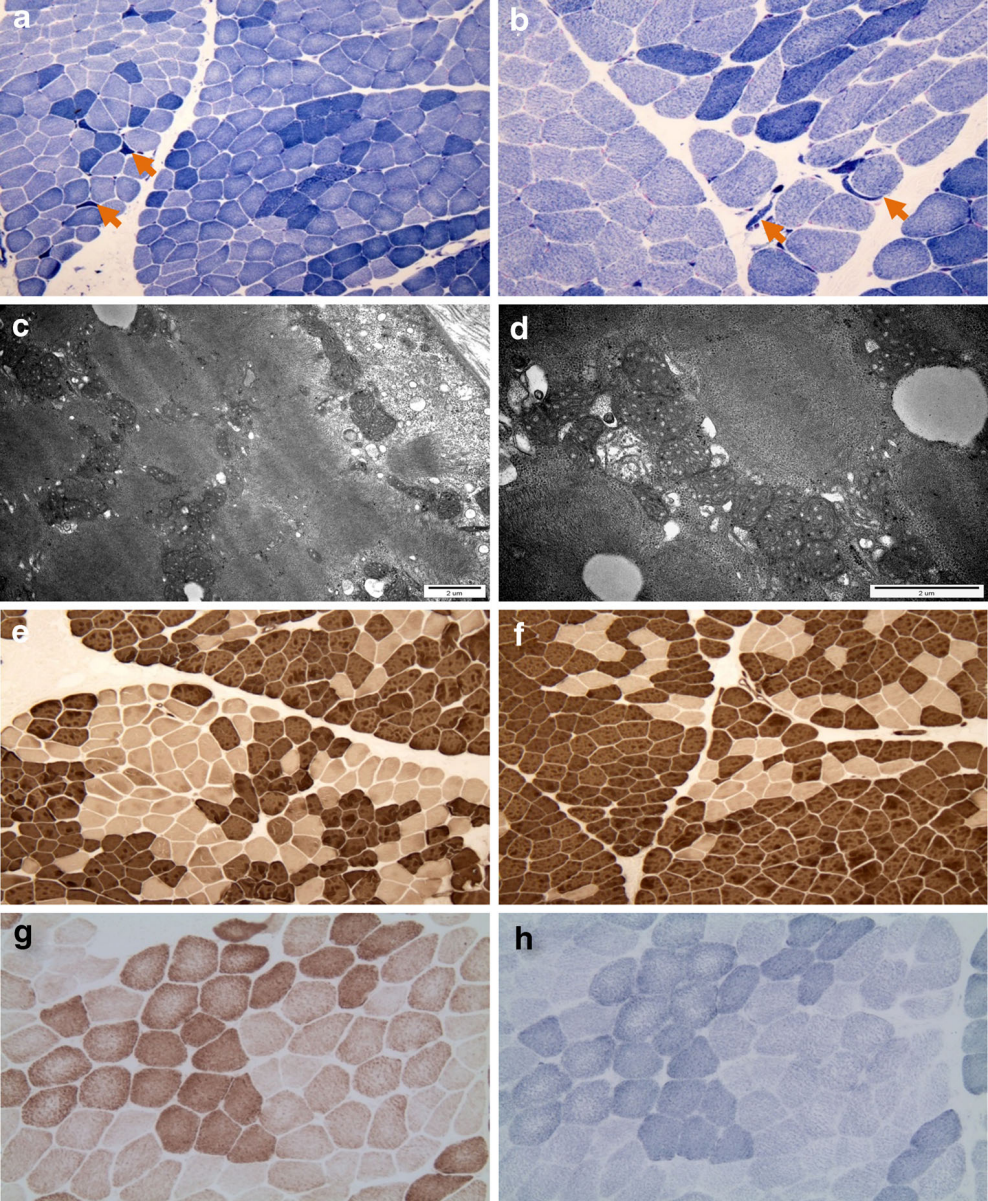
Histopathological characterization of the vastus lateralis muscle (patient II-2). **a**–**b** NADH-TR staining revealed a scattering of small, angulated, or elongated myofibers with condensed positive reactions (*arrows*). **c**–**d** Electron micrographs revealed degenerating myofibers containing diffusely scattered autophagic vacuoles, distorted myofibrillar arrangement, abnormal membranous structures, and aggregated enlarged mitochondria with swelling, concentric cristae, and dense inclusions. **e**–**f** ATPase staining at pH 9.4 revealed grouping of type 1 and type 2 myofibers. **g** Cytochrome oxidase (COX) staining did not show COX-negative fibers. **h** Increased succinate dehydrogenase staining was not observed. Original magnifications: **a** ×100; **b** ×200; **c** ×20,000; **d** ×40,000; **e** ×100; **f** ×100; **g** ×200; and **h** ×200

Biopsy examination revealed variations in the size and shape of fibers, characterized by small, scattered, angulated degenerating myofibers (arrows), including abnormal condensed NADH-tetrazolium-positive cells (Fig. 3a, b). Electron microscopic examination confirmed the presence of degenerating myofibers with diffusely scattered autophagic vacuoles, distorted myofibrillar arrangement, abnormal membranous structures, and aggregated enlarged mitochondria with swelling, concentric cristae, and dense inclusions (Fig. 3c, d). ATPase reactions revealed groupings of both type 1 and type 2 myofibers across multiple pH concentrations (Fig. 3e, f). No differences were seen following cytochrome oxidase (Fig. 3g) or succinate dehydrogenase (Fig. 3h) staining.

### Nerve biopsy findings

**Fig. 4:**
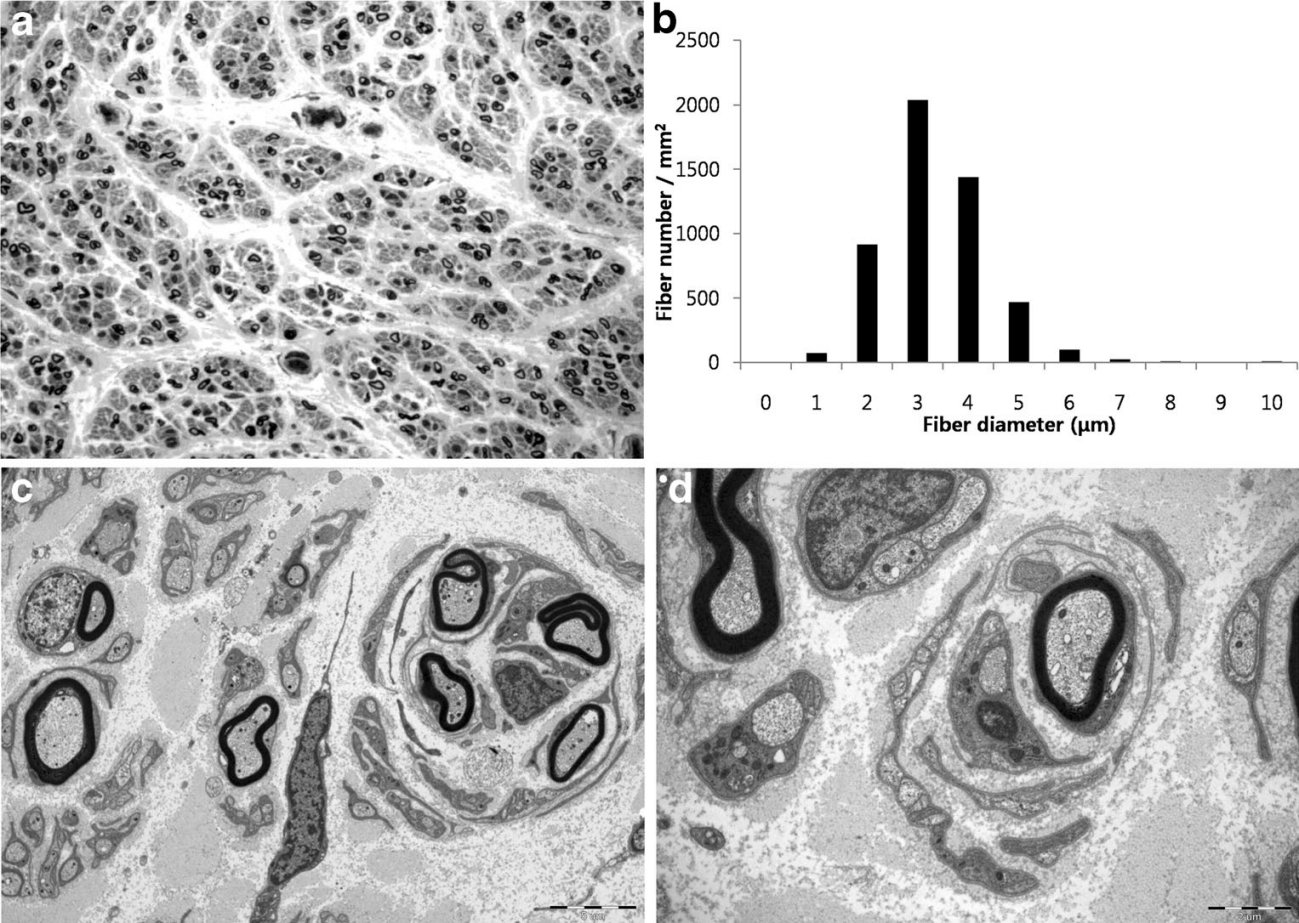
Histopathological characterization of distal sural nerve biopsy (patient II-2). **a** Gray scale image of toluidine blue-stained semi-thin transverse sections revealing an absence of large MFs, along with abundant medium and small-sized MFs (5,101/mm^2^). **b** Histogram showing a unimodal distribution pattern of MFs with diameter <6 μm, representing 97.0 % of total MFs. **c**–**d** Electron micrographs revealed small MFs with excessive folding of myelin and rare axonal clusters. Original magnifications: **a** ×400; **c** ×5,000; and **d** ×12,000

Light microscopic examination of longitudinal and cross sections of nerve fibers showed markedly decreased size of nerve fascicles with marked subperineurial edema and multifocal variable-sized Renaut bodies, which consists of loose EMA-positive spindle cells. Semi-thin transverse sections showed small MFs with almost complete loss of large MFs (Fig. 4a). Remaining MFs counted 5,101/mm^2^ (normal distal sural nerve in 21-year-old male, 10,000/mm^2^). The range and average of diameter of MFs were 1.50–10.13 and 3.87 μm, respectively (The range and average of diameter of MFs in a normal distal sural nerve of a 21-year-old male are 2.2–14.2 and 5.4 μm, respectively). The histogram of diameter sizes showed a unimodal distribution pattern; 19.5 % of the MFs had a diameter less than 3 μm, and 97.0 % had a diameter less than 6 μm (Fig. 4b). The MF% area in this case was 6.46 % (normal sural nerve of a 21-year-old male, 36.5 %). On electron microscopic examination, the remaining small MFs showed occasional excessive folding of myelin with very rare evidence of regeneration (clusters of regenerating fibers, small MFs with collars of Schwann cell processes containing small unmyelinated axons, mimicking a pseudo-onion bulb, or basal lamina onion bulb formation) (Fig. 4c, d). Endoneurial fibroblast proliferation and collagen deposition were evident.

### Brain and lower extremity MRI findings

**Fig. 5:**
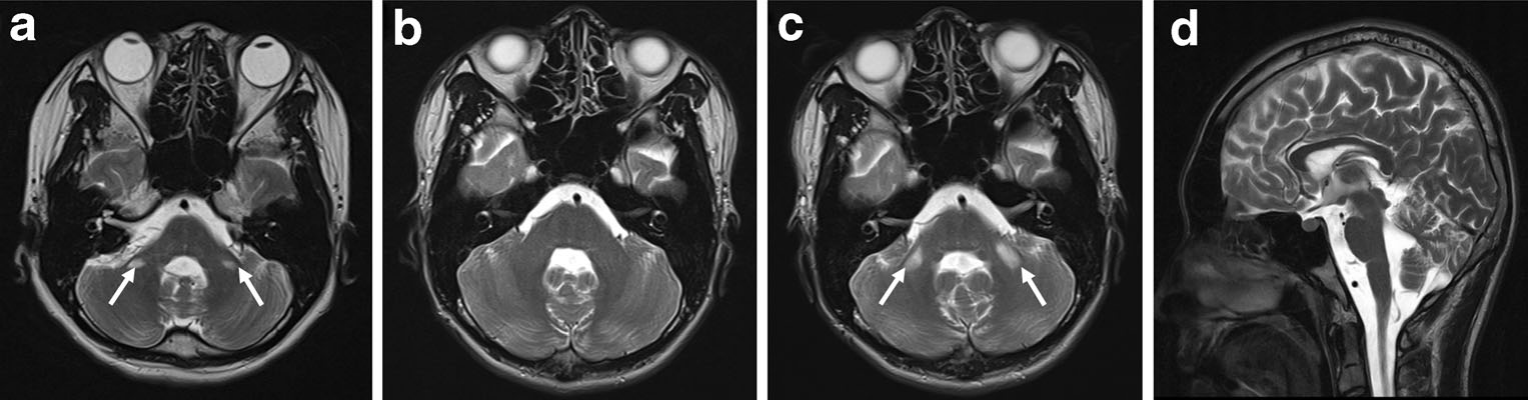
Brain MRI in patient II-1 (**a**) and patient II-2 (**b**–**d**). **a** High-intensity lesions of the bilateral middle cerebellar peduncle (*arrows*) in T2-weighted imaging (patient II-1; 20 years of age). **b** At 17 years of age, no high-intensity lesions were found in the middle cerebellar peduncle of patient II-2. **c** However, a follow-up brain MRI 3 years later revealed bilateral middle cerebellar peduncle lesions (*arrows*). **d** The bilateral cerebellum and brainstem were atrophied, with prominent cerebellopontine cisternal spaces, a widened fourth ventricle, and enlarged cerebellar cortical sulci were observed in patient II-2 at 20 years of age

An MRI revealed hyperintense signal abnormalities in the brains of both patients (Fig. 5). In patient II-1, high-signal-intensity lesions of the bilateral middle cerebellar peduncles were observed on T2-weighted images (Fig. 5a). In patient II-2, similar lesions were not found at the age of 17 years (Fig. 5b); however, a follow-up study performed 3 years later revealed bilateral symmetric high-signal-intensity lesions in the middle cerebellar peduncle (Fig. 5c), with a very similar appearance to those observed in patient II-1. Cerebellum and brainstem atrophy with prominent cerebellopontine cisternal spaces, a widened fourth ventricle, and an enlarged cerebellar cortical sulci were noted in both patients (Fig. 5d). However, the cerebral hemisphere and basal ganglia were normal in both patients, without evidence of atrophy or parenchymal changes.

**Fig. 6:**
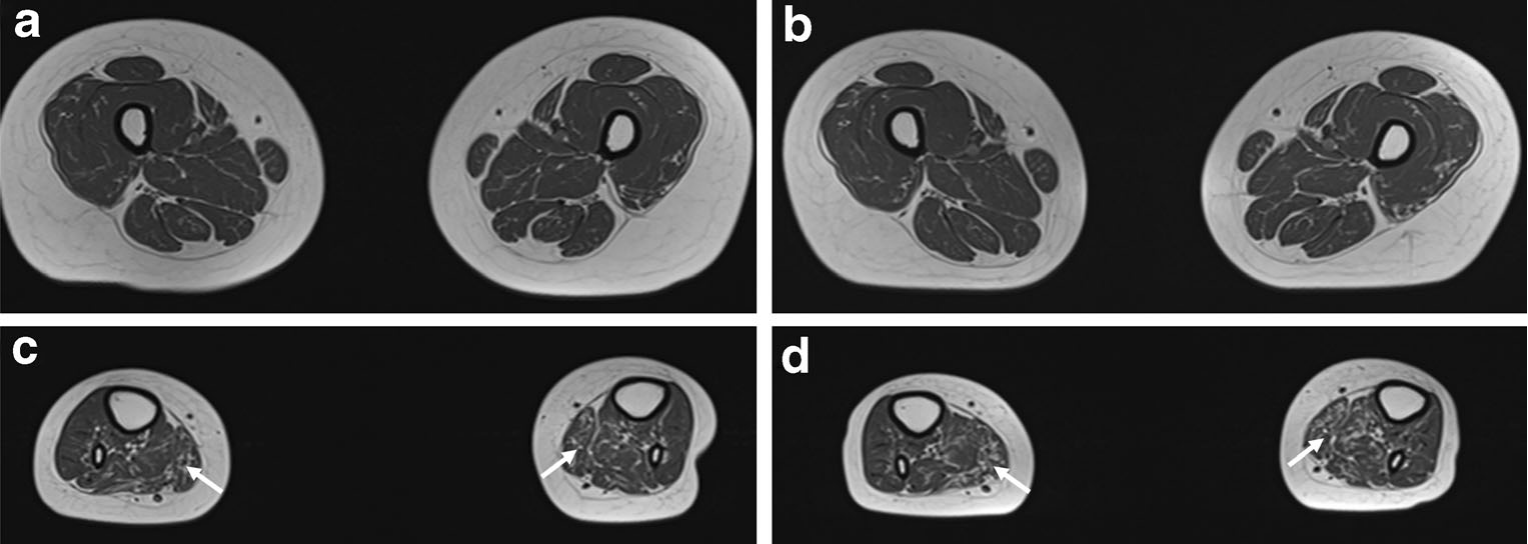
Lower limb MRIs in patient II-1. MRIs of the thigh (**a**, **b**) and lower leg (**c**, **d**) at 20 (**a**, **c**) and 23 (**b**, **d**) years of age. Follow-up studies revealed progression of atrophic changes. **a**, **b** At the thigh level, T1-weighted images demonstrated relatively intact muscle without signal changes or atrophies. **c**, **d** In the lower leg, MRIs revealed moderate involvement of superficial posterior compartment muscles, including the soleus and gastrocnemius muscles (*arrows*); however, anterior and lateral compartment muscles were spared

Relatively intact muscle without abnormal signal changes or muscle atrophy was observed in the thigh (Fig. 6a, b); however, both patients exhibited hyperintense signal abnormalities with muscle atrophy in the calf (Fig. 6c, d). T1-weighted images demonstrated muscle atrophy with signal changes in the superficial posterior compartment, including the soleus and gastrocnemius muscles (arrows). Together, these findings indicate that the distal muscles are impaired more severely than the proximal muscles.

## Discussion

Mutations in *C10orf2* are associated with autosomal recessive IOSCA, hepatocerebral syndrome, and adPEO. Mutations cluster exclusively within the primase, linker, and helicase domains, with the majority of recessive phenotypes occurring as a result of mutations in the helicase domain [17]. In accordance with this mode of action, the two patients described here harbor novel compound heterozygous mutations in the helicase domain (p.T487I and c.1485-1G>A) resulting in phenotypes characteristic of IOSCA. Mutations adjacent to residue T487 have been associated with both dominant and recessive phenotypes: T405I, L456V, R463W, and Y508 mutations cause IOSCA [6, 7, 18], while W474C, A475P, F478I, E479K, and F485L cause PEO [19]. The C1485-1G>A mutation may result in a shorter protein, thereby resembling Twinky, a 582-amino-acid protein terminating with four unique residues. Although this study contained a small number of patients, the identified mutations were clearly validated by Sanger sequencing as well as co-segregation in the kindred (Fig. 1). In addition, neither apparent causative mutation was identified in 648 Korean control samples (Table 2).

The Twinkle helicase protein, encoded by *C10orf2*, localizes to mitochondrial nucleoids and functions in collaboration with DNA polymerase gamma. Mutations within this protein negatively affect the integrity of mtDNA. Deletions of mtDNA are typically associated with adPEO and have not been reported in IOSCA patients [20, 21]. The identification of mtDNA deletions in this study suggests that compound mutations in the helicase domain affect *C10orf2* activity in a recessive manner.

Although a few IOSCA patients have been described in Finland, only three cases have been reported elsewhere, with no cases reported in Asian populations [5–7, 10, 22, 23]. Axonal sensory neuropathy is thought to be the most important pathological feature of IOSCA; however, this report represents the first description of an IOSCA patient harboring motor neuropathy. Before referral to our clinic, the two patients described in this report were first diagnosed with hereditary motor and sensory neuropathies (HMSN) due to sensory and motor neuropathy and moderate to severe foot deformities including high arches. Based upon these initial observations, we believe that the phenotypic spectrum of IOSCA may be wider than reported previously. Screening patients for *C10orf2* mutations may be beneficial for patients with sensorimotor polyneuropathy, particularly HMSN patients who present with symptoms of spinocerebellar ataxia.

Histopathological analysis of the muscle biopsy revealed myopathies harboring small, angulated, or elongated degenerating myofibers containing aggregated, enlarged mitochondria with concentric cristae. High ratios of lactate/pyruvate were also observed. Although there was no evidence of ragged red fibers by Gomori’s trichrome staining, or increased SDH or NADH, the presence of scattered degenerating myofibers with abnormal mitochondria suggests an association with mtDNA deletions.

Brain MRI findings from both IOSCA patients revealed high-intensity lesions of the middle cerebellar peduncles. In patient II-2, no lesions were observed at 17 years of age; however, clear bilateral cerebellar lesions were evident by age 20 years, consistent with observations of more severe ataxia. These results suggest a correlation between the progression of the clinical ataxia and the increasing severity of degenerative IOSCA. Middle peduncular lesions may be useful as a prognostic biomarker of IOSCA. MRI analysis of the lower extremities showed muscle atrophy with hyperintense signal changes in the lower leg muscles of both patients. However, signal intensities were normal in the thigh muscles, consistent with the hypothesis of length-dependent axonal degeneration.

In conclusion, we identified novel compound heterozygous mutations c.1460C>T (p.T487I) and c.1485-1G>A in *C10orf2* as the underlying cause of IOSCA combined with sensorimotor polyneuropathy and myopathy. This study suggests greater variability in the clinical spectrum of IOSCA than reported previously and that screening of *C10orf2* may be helpful in diagnosing IOSCA, especially in patients presenting with symptoms of axonal sensorimotor polyneuropathy and myopathy.

## Electronic supplementary material

Below is the link to the electronic supplementary material.

Supplementary Table 1 (DOC 32 kb)

## Abbreviations

IOSCA: Infantile-onset spinocerebellar ataxia
adPEO: Autosomal dominant progressive external ophthalmoplegia
CMT: Charcot–Marie–Tooth disease
MFs: Myelinated fibers
WES: Whole exome sequencing
mtDNA: Mitochondrial DNA
FDS: Functional disability scale
CMTNS: CMT neuropathy score
HMSN: Hereditary motor and sensory neuropathy
NCSs: Nerve conduction studies
MNCVs: Motor nerve conduction velocities
CMAPs: Compound muscle action potentials
SNCVs: Sensory nerve conduction velocities
NADH-TR: NADH-tetrazolium reductase
SDH: Succinate dehydrogenase
COX: Cytochrome oxidase
ATPase: Adenosine triphosphatase
