# Supplementary material for: Recessive C10orf2 mutations in a family with infantile-onset spinocerebellar ataxia, sensorimotor polyneuropathy, and myopathy
Source: Neurogenetics. 2014 May 10;15(3):171–82. doi: 10.1007/s10048-014-0405-1 (PMC4102772; doi:10.1007/s10048-014-0405-1)
Supplement: Supplementary file 1 — (DOC 32 kb) [file 10048_2014_405_MOESM1_ESM.doc]

| **Supple. Table 1.** Compound heterozygous variants identified through filtering step | | | | | | | | | | |
| --- | --- | --- | --- | --- | --- | --- | --- | --- | --- | --- |
| genomic positions (hg19) | ref_base | alt_base | hom_het | snp_quality | tot_depth | alt_depth | exon | gene | annotation | controls |
| Chr10:102749617 | C | T | het | 49 | 43 | 16 | exonic | *C10orf2* | NM_021830:exon2:c.C1460T | 0/248 |
| Chr10:102750192 | G | A | het | 61 | 93 | 49 | splicing | *C10orf2* | NM_021830:intron3:c.1485-1G>A | 0/248 |
| Chr19:55143632 | C | T | het | 172 | 256 | 124 | exonic | *LILRB1* | NM_006669:exon6:c.C605T | 5/248 |
| Chr19:55145125 | C | A | het | 74 | 23 | 12 | exonic | *LILRB1* | NM_006669:exon9:c.C1298A | 7/248 |
